# Effect of reduction of sodium content on the microbial ecology of Edam cheese samples

**DOI:** 10.1186/s13568-021-01188-7

**Published:** 2021-02-16

**Authors:** Giuseppina Luzzi, Erik Brinks, Jan Fritsche, Charles M. A. P. Franz

**Affiliations:** 1 Departments of Microbiology and Biotechnology, Max Rubner-Institut, Hermann-Weigmann-Str. 1, 24103 Kiel, Germany; 2 Safety and Quality of Milk and Fish Products, Max Rubner-Institut, Hermann-Weigmann-Str. 1, 24103 Kiel, Germany

**Keywords:** Dairy microbiology, Metagenomics, Food fermentation, Food reformulation, Salt reduction, Cheese

## Abstract

Sodium intake is a major risk factor for non-communicable diseases. Consequently, reformulation of cheeses such as Edam to contain less sodium may contribute to lowering disease risk. However, sodium is essential for cheese manufacture, influencing starter culture bacteria activity and abundance during fermentation. This study aimed to assess the microbial diversity of reformulated Edam cheese samples with a reduced sodium content using culture-independent technique. The microbial diversity of samples produced using simple sodium reduction, as well as by substituting salt with a mineral salt compound containing potassium, were analysed in comparison to regular control Edam samples during manufacture and the subsequent 6-week ripening period using 16S rDNA metagenomics. In addition, a challenge test using *Listeria* (*List.*) *innocua* as a surrogate species for *List. monocytogenes* was performed. Reducing sodium content did not influence the microbiological composition of reformulated samples in comparison to that of regular samples. The starter culture bacteria dominated the microbial diversity and no increase in spoilage or potentially pathogenic bacterial growth was detected, including that of *List. innocua*. From a microbiological perspective, it can be concluded that lowering sodium content in Edam samples without affecting the microbial composition is achievable through simple sodium reduction and through implementation of a mineral salt replacement approach.

## Introduction

The use of salt to inhibit microbial growth is a widely used food preservation method. In cheese manufacture, salt protects cheese against pathogens and spoilage bacterial growth (Guinee and Fox 2017). Although sodium, generally consumed as sodium chloride (NaCl; table salt), is essential for important functions of the human body, its excess consumption is a risk factor for non-communicable diseases (NCDs) including coronary heart disease and hypertension (Dötsch et al. 2009; Domnariu et al. 2013). Considering that NCDs contribute to nearly 50% of the global disease burden and are responsible for up to 60% of human mortality worldwide, a reduction of sodium intake could help lower NCD risk (World Health Organization 2014).

In Germany, 10% of dietary sodium consumption stems from cheese and other dairy products (Max Rubner-Institut 2008). Therefore, reducing the salt content of cheese could contribute to lowering sodium intake in the population. However, as sodium has multiplex functions in the production and ripening of cheese, there are many factors to be considered when reformulating cheese. Of particular microbiological importance is the role of salt in lowering the water activity levels and suppressing bacterial spore germination, both of which protect the cheese against pathogen growth and premature spoilage (Smoot and Pierson 1982; El-Bakry 2012). Lactic acid bacteria (LAB), added as mixed starter cultures during cheese manufacture, have a relatively higher salt tolerance than other bacteria, allowing these to thrive in the milk matrix and produce the desired properties of the cheese (Doyle and Glass 2010; Institute of Medicine 2010; Guinee and Sutherland 2011).

Edam cheese is a semi-hard, brine-salted cheese generally produced using mixed mesophilic DL starter LAB cultures (van Hoorde and van Landschoot 2013; Fox et al. 2017). The final salt content is commonly between 2 and 2.4% NaCl (corresponding to ca. 0.8–0.9% Na) (Ayyash et al. 2013; Guinee and Fox 2017). The brining process of Edam cheese involves immersing the pressed curds in a salt solution, whereby the salt content of the cheese is determined by the salt concentration in the brine and the brining time (Guinee 2004). Two strategies for reformulating cheese to contain less salt are by simply lowering the salt applied, as well as a by partially substituting the sodium. In the former approach, the salt content in the brine is simply reduced, and in the latter, mineral salt mixtures are implemented to substitute part of the NaCl in the brine.

In a previous study dealing with technological and sensory aspects, which was done in parallel to this microbiological investigation, Hoffmann et al. (2020) showed that it was possible to lower the sodium content of Edam cheese samples to < 0.4% sodium (equivalent to < 1% NaCl) without affecting the microbial quality, by either simply reducing salt concentration or by partially substituting sodium chloride with mineral salts. Overall, a 30% sodium reduction could be obtained by sodium chloride reduction, while a 50% sodium lowering could be achieved by partial sodium replacement with a potassium (K) containing mineral salt mixture (Hoffmann et al. 2020). Using a culture-dependant microbiological method, i.e. plate counting, the authors found that the starter culture bacteria showed similar growth kinetics in both the simple salt reduction and mineral salt replacement cheese samples, demonstrating typical starter culture growth patterns in Edam cheese samples during fermentation and ripening.

In contrast to the traditional, culture-dependant microbial analyses of starter culture behaviour in Edam cheese samples with reduced sodium content presented by Hoffmann et al. (2020), this study aimed to use culture-independent methods to gain insight into the microbial diversity of the same reformulated Edam samples, by way of metagenomics based on amplicon (16S rDNA) sequencing. The aim of this study was to assess if sodium reduction approaches would affect the microbial diversity of reformulated cheese samples with reference to the starter cultures, as well as the autochthonous bacteria present in milk.

## Materials and methods

### Cheese production and sampling

Raw milk from the dairy research farm (Schädtbek, Germany) of the Max Rubner-Institut in Kiel, Germany was used for cheese sample production. The following mixed LAB starter cultures, obtained from Chr. Hansen (Nienburg, Germany), were utilised concurrently for all Edam manufacture experiments: F-DVS CR-550 [*Lactobacillus* (*Lb*.) species and *Lactococcus (Lac.) lactis* subspecies]; F-ES Easy-Set FLORA™ C-1060 [*Lac*. *lactis* subsp. *lactis*, *Lac*. *lactis* subsp. *lactis* biovar diacetylactis, *Lac*. *lactis* subsp. *cremoris* and an unspecified *Leuconostoc* species]; F-DVS CR-BUTTERY01 [*Lac. lactis* subsp. *lactis, Lb. rhamnosus* (currently reclassified as *Lacticaseibacillus rhamnosus*) and *Lb. paracasei* (currently reclassified as *Lacticaseibacillus paracasei*) (Zheng et al. 2020)]; and F-DVS LH-32 (*Lb. helveticus*).

The milk was pasteurised and subsequently Edam cheese samples with a lowered sodium content were produced using both a straightforward sodium decrease and mineral salt substitution applying the commercial mineral salt product sub4salt^®^ (containing NaCl, KCl and Na-gluconate; Jungbunzlauer Ladenburg GmbH, Ladenburg, Germany), alongside a regular control Edam sample as described in Hoffmann et al. (2020).

Samples for 16S rDNA metagenomic analyses were taken at five sampling stages during manufacture and subsequent ripening of the Edam samples with reduced sodium content: from the cheese milk directly following inoculation of the pasteurised milk with all four starter cultures, from the milk curd immediately prior to curd pressing, and from the cheese samples after one, three and six weeks of ripening at 13 °C. Each cheese production experiment was performed in independent triplicates. For analysis, 100 mL of inoculated milk, and 10 g of curd and cheese samples were used, respectively.

In an additional cheese production experiment, also performed in triplicate, the milk for cheese production was inoculated with 1 × 10^5^ cfu/mL of *Listeria (List.) innocua* together with the starter culture bacteria. In these challenge tests, *List.* *innocua* was used as a non-pathogenic representative for *List. monocytogenes*. This bacterium was previously suggested by the ‘European Union Reference Laboratory for *Listeria monocytogenes*’ as a suitable substitute microorganism, as it shows similar growth characteristics (European Union Reference Laboratory for *Listeria monocytogenes* 2019). The aim of these challenge tests was, therefore, to determine whether a reduction of sodium content would influence the risk of *List. monocytogenes* contamination.

### Sample preparation and genomic DNA extraction

Inoculated milk samples (100 mL) were first subjected to centrifugation at 6000×*g* (30 min, 10 °C) using a Heraeus Multifuge (Thermo Fisher Scientific, Waltham, USA; Rotor 75002005), after which the supernatant was removed. The resulting cell pellets were kept at − 20 °C. Curd and cheese samples were prepared for DNA isolation by blending 10 g of sample with 90 mL of pre-warmed 2% (w/v) sodium citrate buffer (Merck, Darmstadt Germany) in a BagMixer^®^ lab blender (Interscience for Microbiology, Saint Nom, France) for 2 min at maximum speed using a BagFilter^®^ 400 P lab blender bag (< 250 μm lateral filter). The filtered supernatant was again centrifuged at 6000×*g* (30 min, 10 °C). After removal of the supernatant, the pellets were kept at − 20 °C until further processing.

For isolation of genomic DNA, the method published by Luzzi et al. (2020) was modified to suit DNA isolation from inoculated milk, curd and cheese samples. For this, the pelleted samples were defrosted at ambient temperature and resuspended in 1 mL of the lysis buffer used by Luzzi et al. (2020). Next, to each sample 0.4 g of sterile 0.1 mm Zirconium/glass-beads^®^ (Carl Roth, Karlsruhe, Germany) were added and samples were homogenised for 5–10 min at 80 rpm using an Intelli-Mixer RM-2 M (ELMI, Calabasas, CA, USA) bead-beater set at the U2-mode until homogenous. The samples were incubated at 70 °C with shaking at 400 rpm (Eppendorf Thermomixer, Hamburg, Germany) for 15 min. Next, the samples were centrifuged (9600×*g* for 5 min at 4 °C) using a Heraeus Fresco21 (Thermo Fisher Scientific, Waltham, MA, USA) centrifuge. The supernatant was centrifuged repeatedly under the same conditions until a clear lysate was obtained. To increase the final genomic DNA yield, 300 μL of fresh lysis buffer was added after the clear lysate had been harvested, and the homogenisation steps using Zirconium/glass-beads^®^, heat treatment and centrifugation were repeated once as described above.

A 10% volume of 10 M ammonium acetate (relative to the absolute sample volume; Merck, Darmstadt, Germany) was given to the respective clear lysate samples. These were then placed on ice for 10 min and subsequently centrifuged at 16,200×*g* (10 min, 4 °C). To precipitate the DNA, one volume of 2-propanol (4 °C; Carl Roth, Karlsruhe, Germany), was then added to each sample. After mixing thoroughly, the samples were placed on ice for 45 min. This was followed by centrifugation at 16,200×*g* (15 min, 4 °C). The supernatant was discarded and the nucleic acid-containing pellets were washed with 1 mL of 70% ethanol (Carl Roth, Karlsruhe, Germany). Subsequently, the pellets were dried and resuspended in 100 μL of 10 mM Tris-HCl pH 8.0.

Samples were then further processed using the QIAamp DNA Stool Mini Kit (QIAGEN GmbH, Hilden, Germany) according to the protocol outlined by Luzzi et al. (2020). Briefly, the RNA was first digested using 4 μL of a 10 mg/mL DNAse-free RNase solution (VWR International GmbH, Darmstadt, Germany), after which the proteins were degraded with 30 μL of 20 mg/mL proteinase K solution (AppliChem GmbH, Darmstadt, Germany). The QIAGEN kit’s ‘AL Buffer’ was then added to each sample prior to usage of the QIAamp spin columns to capture and wash the DNA as documented by Luzzi et al. (2020). The concentration of genomic DNA from samples was determined with a Qubit^®^ 3.0 Fluorometer and the Qubit™ dsDNA BR Assay Kit following the kit specifications (Thermo Fisher Scientific, Darmstadt, Germany).

### Library preparation and sequencing

The bacterial composition of inoculated milk and cheese samples was assessed using 16S rDNA amplicon sequencing with an Illumina MiSeq™ sequencer (Illumina Inc., San Diego, CA, USA). Library preparation and sequencing was performed according to Luzzi et al. (2020), using the primers targeting the 16S rRNA gene V3 and V4 regions used by these authors.

### Raw data processing and statistical analysis

For all cheese production experiments at all sampling time points, three biological replicates were assessed. The methods used for raw data processing using the Integrated Microbial Next Generation Sequencing platform for ecology and diversity studies (Lagkouvardos et al. 2016), as well as the methods for statistical analysis using the Rhea pipeline in RStudio (Lagkouvardos et al. 2017) were previously published by Luzzi et al. (2020). *Lactobacillus* and *Lacticaseibacillus* genera are still represented by “*Lactobacillus*” in the taxonomic databases for high throughput sequencing, and were thus not distinguished between in this study. An abundance cut-off of 0.05% was used for all relative bacterial abundance displayed and significant differences in microbial composition between groups were calculated as *p*-values of less than 0.05.

## Results

**Fig. 1:**
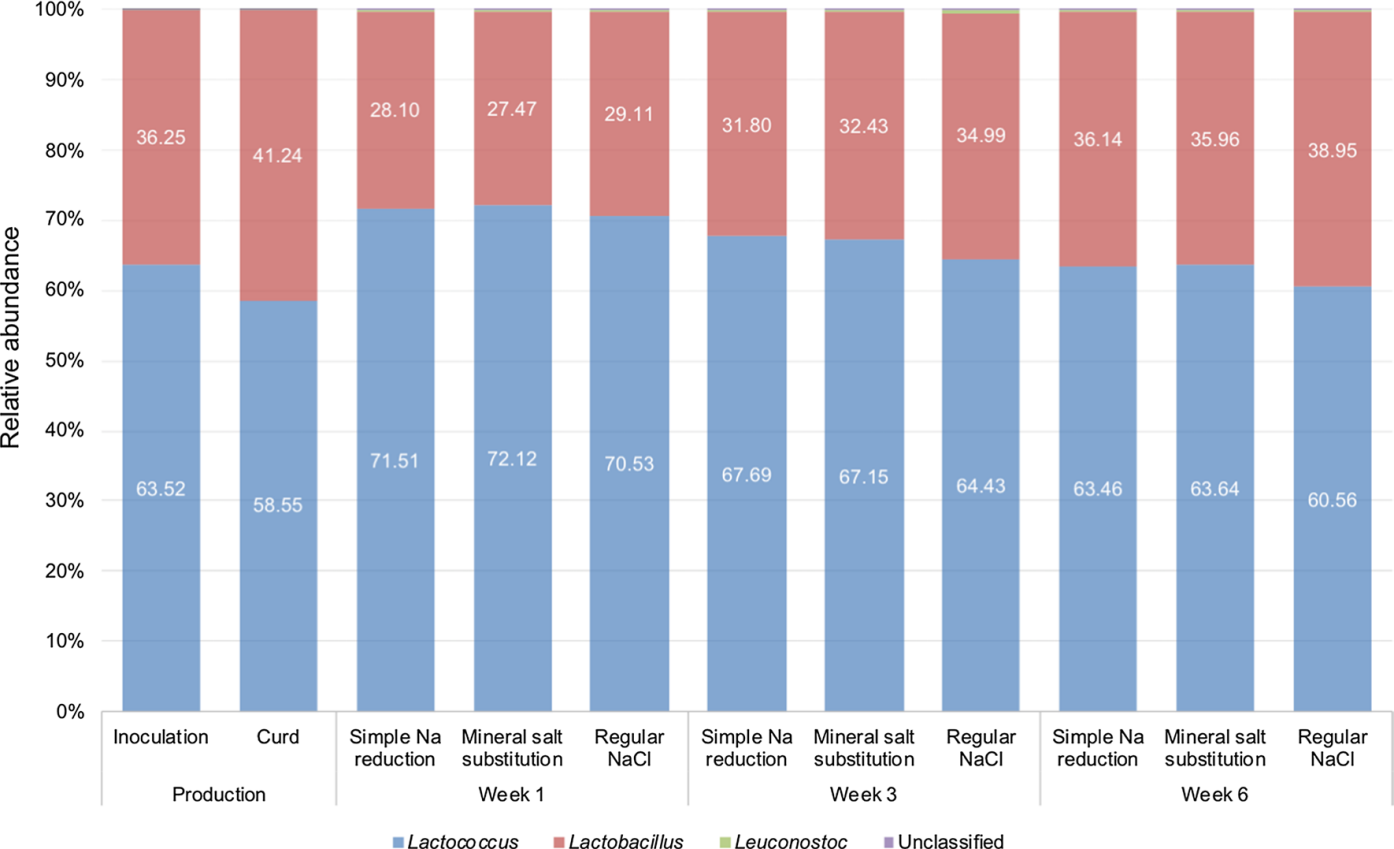
Relative abundance of bacteria (%) in Edam samples with lowered sodium content produced using a simple sodium (Na) reduction and a mineral salt substitution approach, alongside a regular salt (NaCl) Edam control sample. An abundance cut-off of 0.05% was used for all relative bacterial abundance displayed. The adjunct *Leuconostoc* species and unclassified bacteria showed relative abundances of < 0.5% throughout manufacture and ripening. n = 3 biological replicates

To analyse the microbial composition of Edam samples with reduced sodium content, 16S rDNA metagenomics analyses were used. Figure 1 shows the relative abundance of starter culture genera in reformulated Edam samples. Throughout manufacture and subsequent ripening *Lactococcus* and *Lactobacillus* species, which presumptively represented the starter cultures, were dominant components of the microbiota of reformulated Edam cheese. These showed relative bacterial abundances of 58–72% and 36–41%, respectively. The adjunct *Leuconostoc* species, also contained in the starter cultures, stayed beneath 0.5% relative abundance during production and ripening. A small proportion (< 0.2%) of microorganisms in this study were not identified.

**Fig. 2:**
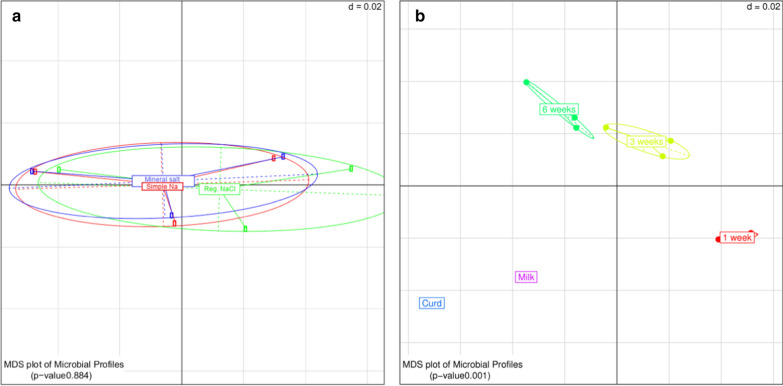
Multidimensional scaling plots displaying **a** the microbial diversity between Edam samples with reduced sodium content (produced using simple Na reduction and mineral salt substitution) and regular NaCl Edam control samples and **b** the microbiological diversity between sampling time points during production and maturation of Edam samples with lowered sodium content and regular NaCl Edam samples. d = dissimilarity of the grid, with a value of 0.02 denoting that the spacing between the grid lines represents a variation of 2% amongst samples. n = 3 biological replicates

Differences in microbiota composition when comparing Edam samples with reduced sodium content and regular Edam samples, as well as cheese samples at different sampling time points, are shown in multidimensional scaling plots in Fig. 2. Statistical analysis indicated no significant difference in bacterial composition of reformulated Edam samples produced by the simple salt decrease and mineral salt substitution methods, when compared to the microbial composition of the control Edam sample (*p* = 0.884; Fig. 2a). However, as the cheese manufacture and ripening progressed, a significant difference in bacterial composition was calculated when comparing the five different sampling time points (*p* = 0.001; Fig. 2b). This shows a significant change, observed most prominently in the relative abundance of *Lactococcus* compared to *Lactobacillus* species at each specific time point, reflecting the expected decline in *Lactococcus* counts and growth of *Lactobacillus* species as ripening progresses.

**Fig. 3:**
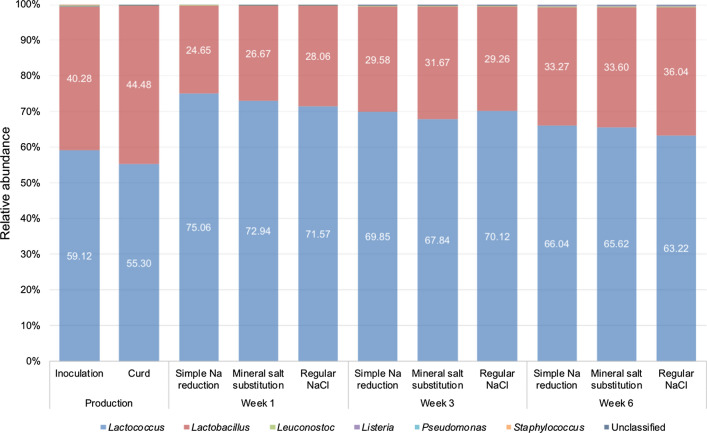
Relative abundance of bacteria (%) in Edam samples that were inoculated with *List.* *innocua* together with the starter bacteria, showing samples with lowered sodium content (produced using a simple sodium (Na) reduction and a mineral salt substitution approach), alongside a regular salt (NaCl) Edam control sample. An abundance cut-off of 0.05% was used for all relative bacterial abundance profiles. The adjunct *Leuconostoc* species and unclassified bacteria displayed relative abundances of < 0.4% throughout manufacture and ripening. n = 3 biological replicates

The microbial composition of Edam cheese samples with lowered salt produced with co-inoculation of *List. innocua* (Fig. 3) showed no noticeable differences to the Edam samples with reduced sodium content as presented above (Fig. 1). Similar to the situation when Edam samples were manufactured without *List. innocua*, the presumptive starter *Lactococcus* and *Lactobacillus* species predominated throughout production and ripening of the cheese samples, represented by relative abundances of 55–75% and 24–45% respectively. At all the sampling time points, the relative abundance of adjunct *Leuconostoc* species was below 0.5%. In these samples, the percentage of unclassified microorganisms was < 0.4%.

**Fig. 4:**
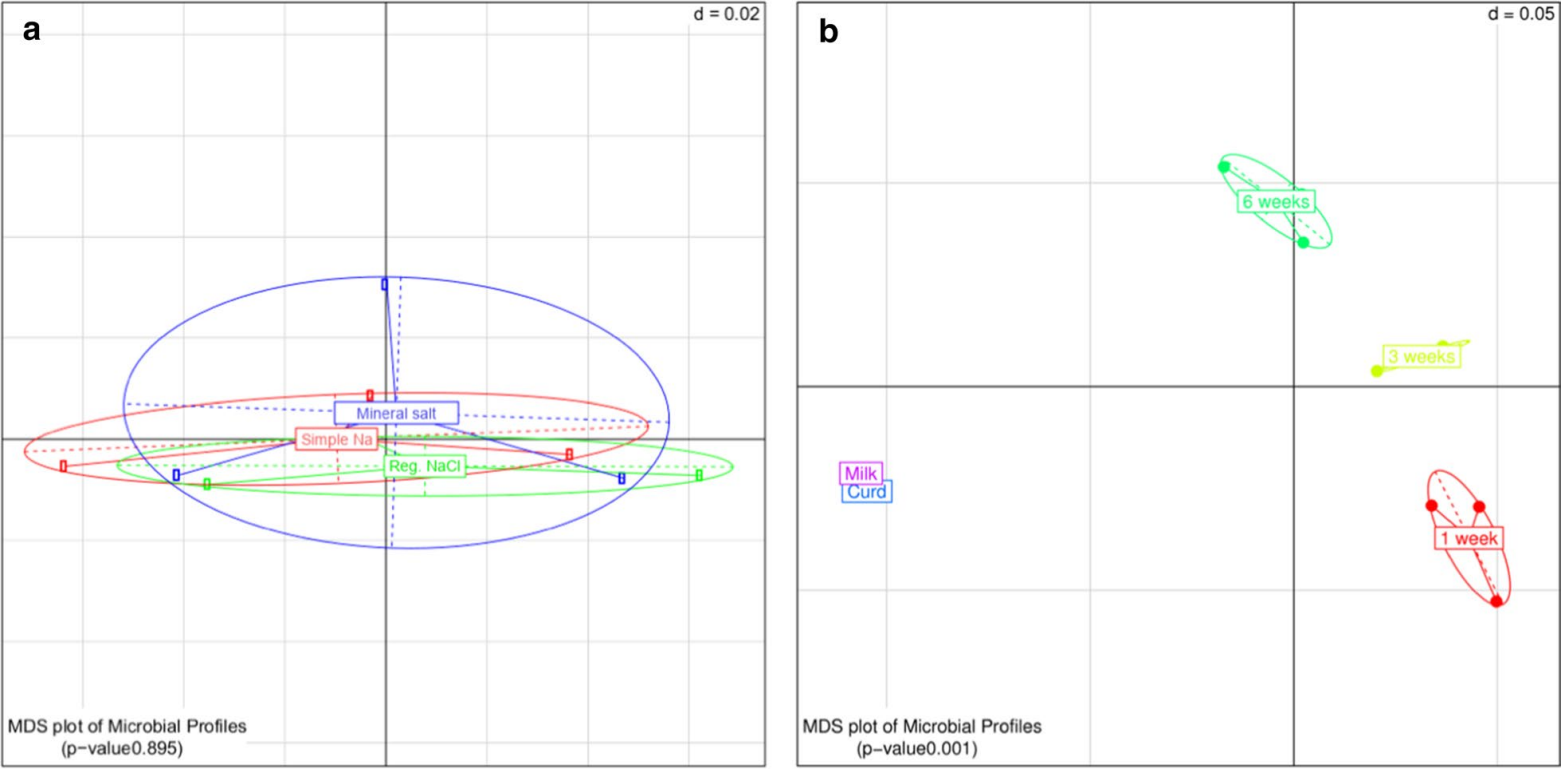
Multidimensional scaling plots displaying **a** the microbial diversity between Edam samples co-inoculated with *List.* *innocua*, produced with lowered sodium content (manufactured using simple Na reduction and mineral salt substitution) and produced as regular NaCl Edam control samples and **b** the microbial diversity between sampling time points during production and maturation of Edam samples with reduced sodium content and regular Edam samples co-inoculated with *List.* *innocua*. d = dissimilarity of the grid, with a value of 0.05 denoting that the spacing between the grid lines represents a variation of 5% amongst samples. n = 3 biological replicates

Statistical analysis of cheese samples in the experiment in which *List. innocua* was inoculated together with the starter bacteria during the course of production was done using the Rhea pipeline and was also visualised through multidimensional scaling plots (Fig. 4). These analyses also showed that the microbial composition of the reformulated Edam samples did not differ significantly to that of the control Edam samples (*p* = 0.895; Fig. 4a). As also observed previously with the cheese samples produced without addition of *List.* *innocua*, the composition of microbiota changed in a statistically significant way as ripening progressed (*p* = 0.001; Fig. 4b). This indicated that the microbial composition of all samples taken at a single sampling time point (including both reformulated and control samples) was different to the microbiota composition of all samples at the next time point, reflecting typical expected LAB growth behaviours during cheese ripening. Although the *List.* *innocua* strain was co-inoculated at a level of 1 × 10^5^ cfu/mL, the minimal relative abundance of DNA from this species could only be detected after three (0.1%) and 6 weeks (0.2–0.3%) of ripening.

## Discussion

In this study, the cheese microbiota of Edam samples with reduced sodium content was assessed during the course of cheese production and ripening using 16S metagenomics. Two sodium reduction strategies were investigated: simply lowering salt concentration or partially replacing sodium chloride with mineral salts. Statistical analyses of the 16S rDNA metagenomics data showed that the microbial diversity of reformulated Edam samples with reduced sodium content and control samples at any of the five sampling time points during production and ripening were not significantly different. These culture-independent results indicate that lowering the sodium content did not appear to influence the behaviour of the implemented LAB starter cultures during the ripening process. These results are supported by those previously reported (Hoffmann et al. 2020), in which culturing methods were used to follow the viable counts of starter *Lactococcus*, *Lactobacillus* and *Leuconostoc* strains in the same Edam samples with reduced sodium content during manufacture and ripening. In that study, it was furthermore demonstrated that the presumptive starter culture species showed typical bacterial growth progressions in both cheese samples with lowered sodium content as well as in the control Edam samples at all five sampling time points. The current 16S rDNA metagenomics study builds on the Hoffmann et al. (2020) data and provides more detailed information on the microbial ecological composition of Edam samples with lowered sodium content in relation to time.

The *Lactococcus* species starter clearly was the predominant component of the microbiota of all Edam samples with reduced sodium (55–75%), followed by the probable starter and adjunct *Lactobacillus* strains which still constituted a sizeable component of 22–45%. The Edam samples in this investigation were ripened for only 6 weeks. This could explain why the bacterial abundance of non-starter LAB strains was extremely low, as these bacteria occur at higher levels only in later stages of ripening (Blaya et al. 2017). The adjunct *Leuconostoc* cultures that exhibited the expected growth kinetics in culture-dependent determinations, as previously demonstrated by Hoffmann et al. (2020), were only detected below 1% relative abundance in the 16S rDNA metagenomics analyses. Although this may be a potential consequence of primer bias affecting this result, a further and more likely reason is the high relative abundances of *Lactococcus* and *Lactobacillus* starter culture species. These were added at 1–2.5 log cfu/mL higher than the *Leuconostoc* culture, as was previously shown using culture-dependent methods (Hoffmann et al. 2020), and hence an overshadowing of the relative abundance of *Leuconostoc* cultures in the 16S rDNA metagenomics profiles seems to be prominent. In cheese samples with ripening times longer than 6 weeks, the bacterial counts of adjunct cultures such as *Leuconostoc* species are anticipated to increase as ripening progresses (Gobbetti et al. 2015).

Salazar et al. (2018) reported on the microbiota of commercial Gouda cheese, analysing differences in microbiota occurring according to spatial distribution within the cheese wheels and length of cheese ripening using metagenomics. They also showed that although samples taken from the core showed some variation in the distribution of microbial abundance, all samples were dominated by *Lactococcus* species (46–55%) and these could reasonably be attributed to the starter cultures. This resembles our results on the bacterial abundance in Edam samples with decreased sodium content in this investigation. However, in the study of Salazar et al. (2018), the percentage of *Lactobacillus* species abundance was lower (3–5%) in commercial Gouda cheese and a high percentage of unidentified members of the *Bacillaceae* family (40%), were observed, which was not the case in this investigation. These discrepancies may be partly attributed to the diverse microbiota of raw milk prior to pasteurisation (Quigley et al. 2013) or possibly in the different production environments. Furthermore, Salazar et al. (2018) showed that the time used for maturation had a tremendous influence on the bacterial species richness in commercial Gouda, suggesting that longer ripening times of Edam with reduced sodium content may also result in increased microbial diversity in the end product.

Quigley et al. (2012) also used amplicon (16S rRNA gene) sequencing to analyse the microbiota of artisanal cheeses and they also found *Lactococcus* species to predominate in semi-hard cheeses similar to Gouda and Edam cheese, with the LAB comprising 84% *Lactococcus*, 7.3% *Lactobacillus* and 0.5% *Leuconostoc* species. These studies showed furthermore that cheeses which are produced with brief ripening periods, even though differing in bacterial diversity and abundance, have in common that the bacterial diversity during the first ripening months is driven by the development of the starter bacteria combinations, especially the *Lac.* *lactis* starters.

In the challenge test in which Edam cheese with reduced sodium content was co-inoculated with the *List.* *monocytogenes* substitute *List.* *innocua*, Hoffmann et al. (2020) demonstrated that the pathogen surrogate did not influence production or ripening of either the reformulated Edam varieties or regular Edam samples, and that *Listeria* species survival between salt-reduced and standard cheese samples did not differ. These results were confirmed by the current 16S metagenomics study, whereby it was possible to uncover the minimal relative abundance of *Listeria* species in sodium-reduced and regular Edam samples after 3 weeks and after 6 weeks of ripening. Studies investigating *List.* *monocytogenes* survival in sodium-reduced Cheddar cheese have shown similar results (Shrestha et al. 2011; Hystead et al. 2013). A comparable study by Hystead et al. (2013) examined sodium reduction, in addition to KCl supplementation on the behaviour of *List.* *monocytogenes* in Cheddar cheese. Different time points of post-processing contamination were analysed, and it could be shown that lowering sodium content up to 50% and a 1:1 substitution of NaCl with KCl did not affect *List.* *monocytogenes* survival. The pathogen did, however, survive better when inoculated at later time points after processing. Considering the similarities of the study by Hystead et al.(2013) with that of Hoffmann et al. (2020) and the current study, it is possible that contamination of sodium-reduced Edam with *Listeria* species at a later stage during ripening rather than during production could lead to its increased growth in Edam with reduced sodium content.

Furthermore, in the study of Hoffmann et al. (2020) the growth of enterobacteria, enterococci, yeasts, moulds or pseudomonads in reformulated, sodium-reduced Edam samples was also tested for after 6 weeks of ripening and demonstrated that the microbiological quality of Edam samples with lower sodium content was not affected by these spoilage and potentially pathogenic organisms. The relative abundance profiles generated through 16S rRNA gene sequencing in the current study confirmed the previous culture-dependent analyses demonstrating an absence of these bacterial species.

In conclusion, the current study was able to demonstrate that lowering the sodium content of Edam cheese samples, whether applied through a simple sodium reduction approach or a mineral salt replacement strategy, did not affect the bacterial composition of the reformulated samples when compared to the regular Edam cheese samples. The microbial diversity did not differ between either varieties of sodium-reduced Edam or the control Edam samples at any sampling time point during cheese production and the 6-week ripening period. The reformulation approaches investigated in this study did not display any effect on the essential growth of the starter LAB central to the production of these fermented dairy product samples. Furthermore, the metagenomics results confirmed previous culture-dependent analyses, showing that the reduction of sodium content in Edam samples did not lead to more growth of spoilage or potentially pathogenic bacteria in the samples.

## Data Availability

The raw 16S rDNA sequencing data have been deposited in the Sequence Read Archive (SRA) with links to BioProject accession number PRJNA660257 in the NCBI BioProject database (https://www.ncbi.nlm.nih.gov/bioproject/).
